# scME: a dual-modality factor model for single-cell multiomics embedding

**DOI:** 10.1093/bioinformatics/btad337

**Published:** 2023-05-23

**Authors:** Bin Zhou, Fan Yang, Feng Zeng

**Affiliations:** Department of Automation, School of Aerospace Engineering, Xiamen University, Xiamen 361102, Fujian, China; Department of Automation, School of Aerospace Engineering, Xiamen University, Xiamen 361102, Fujian, China; Department of Automation, School of Aerospace Engineering, Xiamen University, Xiamen 361102, Fujian, China; Department of Neuroscience, School of Medicine, Xiamen University, Xiamen, Fujian 361005, China; National Institute for Data Science in Health and Medicine, School of Medicine, Xiamen University, Xiamen 361005, China

## Abstract

**Motivation:**

Single-cell multiomics technologies are emerging to characterize different molecular features of cells. This gives rise to an issue of combining various kinds of molecular features to dissect cell heterogeneity. Most single-cell multiomics integration methods focus on shared information among modalities while complementary information specific to each modality is often discarded.

**Results:**

To disentangle and combine shared and complementary information across modalities, we develop a dual-modality factor model named scME by using deep factor modeling. Our results demonstrate that scME can generate a better joint representation of multiple modalities than those generated by other single-cell multiomics integration algorithms, which gives a clear elucidation of nuanced differences among cells. We also demonstrate that the joint representation of multiple modalities yielded by scME can provide salient information to improve both single-cell clustering and cell-type classification. Overall, scME will be an efficient method for combining various kinds of molecular features to facilitate the dissection of cell heterogeneity.

**Availability and implementation:**

The code is public for academic use and available on the GitHub site (https://github.com/bucky527/scME).

## 1 Introduction

Although single-cell RNA sequencing (Hwang *et al.* 2018) has been a powerful tool to investigate the transcriptional dynamics of cells in living things, it is increasingly aware of that cell heterogeneity cannot be fully and correctly determined with the use of only one kind of molecular feature, i.e. CD4+ and CD8+ T-cell subtypes. Moreover, the unimodal dataset is limited to revealing the complex structure of biological information processing, where multiple distinct molecules are cascaded to ensure the correctness of cell fate decisions and functions. Several single-cell multiomics technologies are encouragingly emerging to address these issues by measuring various kinds of molecular features instead of one kind of molecular feature. For example, CITE-seq (Stoeckius *et al.* 2017) can quantify transcripts and cell-surface proteins in the same cells with oligonucleotide-conjugated antibodies. SHARE-seq (Ma *et al.* 2020) and 10× Multiome (Zheng *et al.* 2017, Satpathy *et al.* 2019) can quantify transcripts and chromatin openness in the same cells. Single-cell multiomics bring up new tasks requiring new computational methods to effectively process multimodal information and combine different kinds of molecular features to discover insightful biological events.

A task of fundamental multimodal data processing is joint representation learning of multiple modalities, which aims to embed cells in a low-dimensional space so that one cell and its neighboring cells share similar multimodal features. To obtain a proper joint low-dimensional embedding for a multimodal dataset, one needs to overcome the following challenges. First, single-cell multiomics confront diverse sources and types of noise. Data quality varies vastly across modalities. To address this issue, Weighted Nearest Neighbor (WNN) (Hao *et al.* 2021) estimates the utility of a modality based on the consistency of the information contained in local neighborhoods defined by k-nearest neighboring (KNN). WNN devises a weighted strategy to compute the joint embedding of multimodal data, where results can be impacted by modalities with good information utility. Second, the information provided by multiple modalities is complicated, describing various aspects of cell characteristics. Conceptually, multimodal information can be cataloged into two kinds (Lin and Zhang 2022). One kind of multimodal information is shared information across modalities that can lead to a consistent statement about the state of a cell. For example, if shared information claims that one set of cells is different from the other set of cells, these two sets of cells shall be separated in every modality. The other kind of multimodal information is complementary information that provides distinct cell characteristics in a modality, which are not observable in other modalities. On the one hand, it is difficult to identify shared information and complementary information. On the other hand, it is difficult to combine shared information and complementary information, well balancing the contribution of distinct information to downstream tasks such as clustering and classification.

Accurate identification and combination of shared and complementary information are critical for joint representation learning of single-cell multiomics. The commonly adopted approach is to retain shared information. For example, MOFA+ (Argelaguet *et al.* 2020) uses principal component analysis (PCA) to extract the shared information across various modalities and discard complementary information. To deal with the complexity of multimodal data, totalVI (Gayoso *et al.* 2021) uses a variational autoencoder (Kingma and Welling 2014) instead of PCA to compute the shared information among modalities. Though totalVI is powerful, a method that can effectively distinguish and combine both shared and complementary information, to reveal the variations among cells maximally, is still lacking.

To address the above issue, we designed a factor model involving one common latent variable to describe shared information across modalities and the other latent variables to describe complementary information in each modality. We implemented the factor model with an architecture resembling that of supervised deep generative models, which have been recently recognized as effective factorizing methods to extract information relating to factors (Kim and Mnih 2018). In practice, there are no precise definitions of shared information and complementary information and no means to distinguish them. To tackle this difficulty, we run clustering on each modality at the beginning and use the clustering labels as the indicator of modality-specific information to supervise factorization and parameter estimation. This behaves like self-supervised learning. The proposed factor model is trained by using stochastic variational inference (Hoffman *et al.* 2013) and thus is highly scalable. We hypothesized that the combination of the common latent variable and the distinct information specific to a modality shall provide sufficient information for accurate reconstruction of the observations in all modalities. We conducted a series of experiments to demonstrate that the factor model significantly improves cross-modality data integration and generates joint embedding that is useful for several downstream tasks, including clustering, classification, and data visualization.

## 2 Materials and methods

### 2.1 Supervised deep generative model for factor analysis

**Figure 1.**
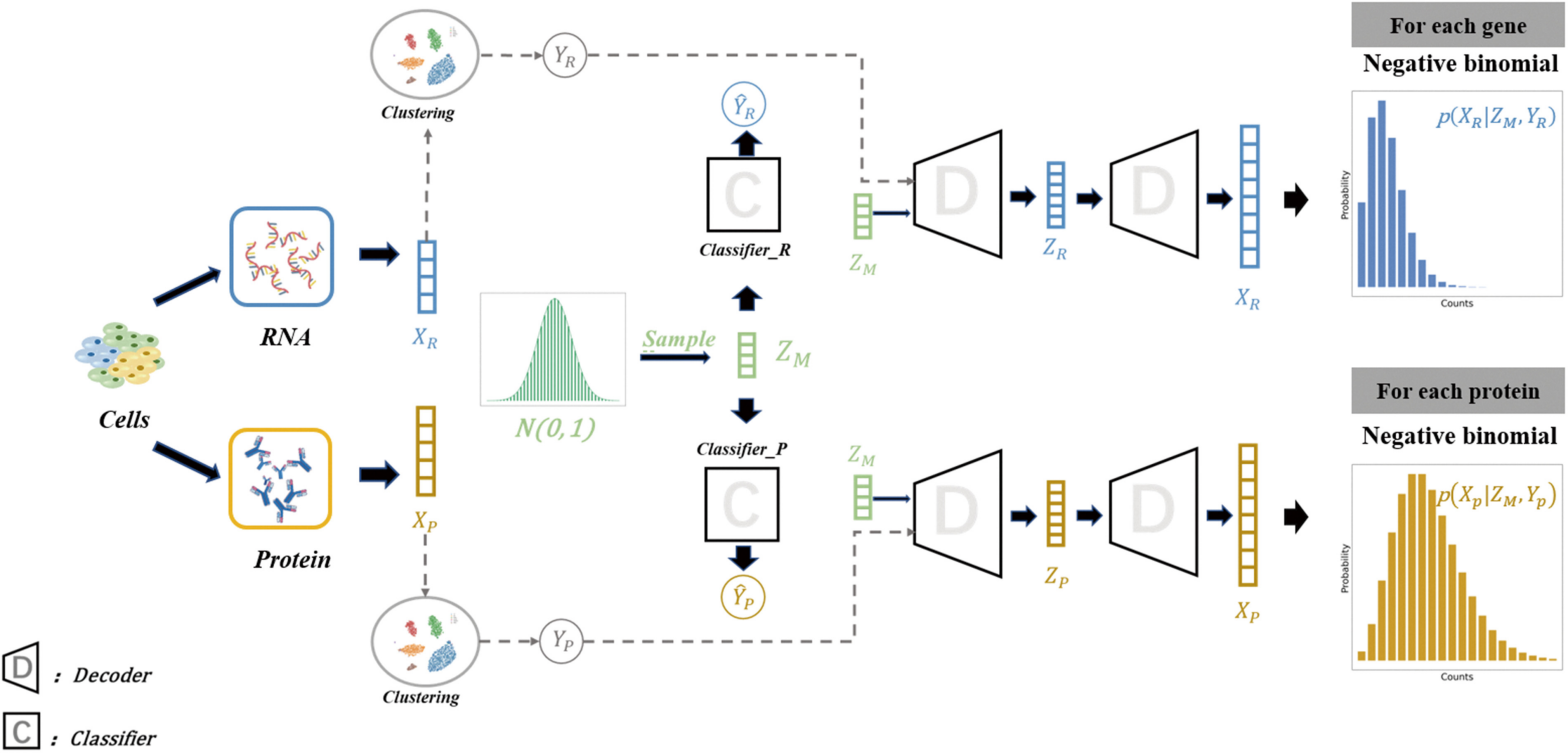
Overview of scME model.

To maximumly utilize multimodal information, the key is to recognize and extract both shared information and complementary information across multiple modalities. In this article, we introduce a dual-modality factor model for the extraction of information on various properties of single-cell multiomics (Figure 1). The proposed dual-modality factor model is derived from the supervised deep generative model. Supervised deep generative models have been broadly applied in the field of single-cell data analysis. In addition to dimensionality reduction and classification, supervised deep generative models can be interpreted as factor models. From the perspective of factor model, supervised deep generative models can be represented as the following generation process controlled by a set of factors. For simplicity, let us consider a factor model with two factors. One of the factors is data label y, which indicates the principal factor affecting the variation of data. The other factor $z_{u}$ is to describe the impact of the resting factor(s) that cannot be characterized or is unknown at the moment. Thus, the complete state of data can be represented by the following equation,


(1) z∼y+zu .

Then, it is to generate a data X from the complete state z as follows,


(2) X∼z#.

In the aforementioned factor model, the data label y is to represent the axis of variation in data to be extracted. After training with the cross-entropy loss, the supervised deep generative models are capable of extracting information regarding the variation of y. Unexplainable data variation can be captured by $z_{u}$. Factor models based on the supervised deep generative models are increasingly used for disentangled representation learning in computer vision. It has been used to successfully disentangle the style factor and the digit factor in the image data of handwritten digits (Paige *et al.* 2017). Here, we extend the model to the factorization of single-cell multiomics.

### 2.2 scME model

Enlightened by the factorization of supervised deep generative learning, we proposed and developed a dual-modality factor model for the analysis of single-cell multiomics. For clarity and simplicity of illustration, we use CITE-seq (Stoeckius *et al.* 2017) throughout the article. For CITE-seq, a cell has two modalities, including the RNA modality and protein modality. First of all, we introduce the general concept of the dual-modality factor model. We define a latent variable $Z_{M}$ to denote the shared information. Additionally, we involve two distinct latent variables $Y_{R}$ and $Y_{P}$ to represent the complementary information of the RNA modality and the protein modality, respectively. Due to a lack of precise definitions regarding shared information and complementary information, it is difficult to factorize the complicated information contained in single-cell multiomics. To overcome the difficulty, we adopt a self-supervised-like strategy. Specifically, we perform Leiden clustering (Traag *et al.* 2019) on the RNA modality and the protein modality at the beginning. Then, the clustering labels of the two modalities are used as $Y_{R}$ and $Y_{P}$ to indicate the modality-specific information. The latent state of the RNA modality can be specified by using the shared information $Z_{M}$ and the complementary information $Y_{R}$ as follows,


(3) ZR∼ZM+YR#.

Similarly, the latent state of the protein modality can be specified by using the shared information $Z_{M}$ and the complementary information $Y_{P}$, that is,


(4) ZP∼ZM+YP#.

After the specification of the latent states, the observations $X_{R}$ and $X_{P}$ of two modalities can be reconstructed with the well-defined generative processes $G_{R}$ and $G_{P}$ as follows,


(5) XR∼GRZRXP∼GPZP#.

The detailed information regarding the latent factors and generative processes are given in the following.

Suppose there is a CITE-seq dataset comprised of n cells, where we denote the RNA modality as $X_{R}=\left\{X_{R,i}\right\}$ and the protein modality $X_{P}=\left\{X_{P,i}\right\}$, 1≤i≤N, respectively. First, we specify the distribution models regarding $Z_{M}$, $Y_{R}$, and $Y_{P}$. The shared information $Z_{M}$ is modeled with a standard normal distribution, where $0\in R^{d}$ is the zero-mean vector and $I\in R^{d\times d}$ is the identity matrix. The number d is the dimension of the low-dimensional embedding space. Since the complementary information are discrete clustering labels, $Y_{R}$ and $Y_{P}$ are modeled with the categorical distribution as follows, and where $1_{R}=\left[1,1,\ldots ,1\right]$ and $1_{P}$ represent the parameters of the non-informative prior Dirichlet distributions over $\theta_{R}$ and $\theta_{P}$. The number of clusters in the RNA modality is $\left|1_{R}\right|=k_{R}$. The number of clusters in the protein modality is $\left|1_{P}\right|=k_{P}$.


(6) ZM∼N0,I#,

(7) θR∼Dirichlet1RYR∼CategoricalθR#,

(8) θP∼Dirichlet1PYP∼CategoricalθP#,

Next, we specify the modality-specific generation process. To generate the observations of the RNA modality, the generation process starts with the generation of the modality-specific latent state $Z_{R}$ followed by a multinomial generation process. The complete generation process of RNA observations is formulated as follows, where $f_{wr}$ and $f_{\mathrm{wdr}}$ are the multiple layer neural networks. Similarly, the generation of the protein observations is formulated as follows, where $f_{wp}$ and $f_{\mathrm{wdp}}$ are the multiple layer neural networks.


(9) $$\begin{array}{l} \mu_{R,i},\Sigma_{R,i}=f_{wr}\left(Z_{M,i},Y_{R,i}\right)\\ \begin{array}{l} Z_{R,i}∼\mathrm{Normal}\left(\mu_{R,i},\Sigma_{R,i}\right)\#\\ \begin{array}{l} {\gamma_{R,i},\alpha }_{R,i}=f_{\mathrm{wdr}}\left(Z_{R,i}\right)\\ X_{R,i}∼\mathrm{NegativeBinomial}(\gamma_{R,i},\alpha_{R,i}), \end{array} \end{array} \end{array}$$

(10) $$\begin{array}{l} \mu_{P,i},\Sigma_{P,i}=f_{wp}\left(Z_{M,i},Y_{P,i}\right)\\ \begin{array}{l} Z_{P,i}∼\mathrm{Normal}\left(\mu_{P,i},\Sigma_{P,i}\right)\#\\ \begin{array}{l} {\gamma_{p,i},\alpha }_{p,i}=f_{\mathrm{wdp}}\left(Z_{p,i}\right)\\ X_{P,i}∼\mathrm{NegativeBinomial}({\gamma_{p,i},\alpha }_{p,i}) \end{array} \end{array}, \end{array}$$

To utilize the scalable variational inference, we design the variational distribution over the latent variables as the following product of factorized distributions,


(11) qϕZM,ZR,ZP  XR,XP=qϕZM  ZR,ZPqϕZR  XRqϕZP  XP#.

The loss function is comprised of two components. The first component is the likelihood that the proposed dual-modality factor model generates the observed data of both two modalities. Due to that the true data distribution is unknown, the data likelihood is approximated by the variational distribution. Therefore, the evidence lower bound (ELBO) can be obtained as follows,


(12) $$\mathrm{ELBO}=E_{q_{\phi }}\left[\log\;p_{\theta }\left(X_{R}|Z_{M},Z_{R}\right)+\log\;p_{\theta }\left(X_{P}|Z_{M},Z_{P}\right)\right]+D_{KL}(q_{\phi }\left(Z_{M}|Z_{R},Z_{P}\right)||p_{\theta }\left(Z_{M}\right))\#+D_{KL}(q_{\phi }\left(Z_{R}|X_{R},Y_{R}\right)||p_{\theta }\left(Z_{R}\right))+D_{KL}(q_{\phi }\left(Z_{P}|X_{P},Y_{P}\right)\left\|p_{\theta }\left(Z_{P}\right)\right.).$$

The other component regards the accuracy of label prediction. For the prediction of RNA cluster labels and protein cluster labels, we use the binary cross-entropy losses (BCE) for two modalities, that is, where $f_{CR}$ and ${\;f}_{CP}$ are two multiple layer neural networks for label prediction, where the last layer adopts the Softmax activation function. The total loss function is defined as.


(13) $$\begin{array}{l} B\mathrm{CE}\left(Y_{R},{\hat{Y}}_{R}\right)=Y_{R}\log{\hat{Y}}_{R}+\left(1-Y_{R}\right)\log\left(1-{\hat{Y}}_{R}\right)\\ \begin{array}{l} {\hat{Y}}_{R}=f_{CR}\left(Z_{M}\right)\#\\ \begin{array}{l} B\mathrm{CE}\left(Y_{P},{\hat{Y}}_{P}\right)=Y_{P}\log{\hat{Y}}_{P}+\left(1-Y_{P}\right)\log\left(1-{\hat{Y}}_{P}\right)\\ {\hat{Y}}_{P}=f_{CP}\left(Z_{M}\right) \end{array} \end{array} \end{array},$$

(14) L=ELBO+BCEYR,Y^R+BCEYP,Y^P#.

We estimate the parameters of the proposed dual-modality factor model using the Adam optimizer and stochastic variational inference (Hoffman *et al.* 2013) algorithm.

### 2.3 Datasets and preprocessing

#### 2.3.1 Datasets

We used four benchmarking datasets to evaluate the performances of the dual-modality factor model and the state-of-the-art methods. The four datasets are CBMC (Linderman *et al.* 2022), BMNC (Stuart *et al.* 2019), BM2 (Luecken *et al.* 2021), and SLN (Gayoso *et al.* 2021), which are generated by CITE-seq. All four benchmarking datasets are also available on the National Center for Biotechnology Information (NCBI) GEO database. The statistics and GEO accession numbers of the four datasets are given in Table 1.

**Table 1.** Statistics and GEO accession numbers of four datasets.

Datasets	Cell size	Species	Protocol	GEO
BMNC	30 672	Homo sapiens	CITE-seq	GSE128639
BM2	120 000	Homo sapiens	CITE-seq	GSE194122
SLN	32 648	Mus musculus	CITE-seq	GSE150599
CBMC	8617	Homo sapiens	CITE-seq	GSE100866

#### 2.3.2 *Data preprocessing, visualization, and clustering*

All datasets are preprocessed following the standard instructions of the Seurat protocol (Butler *et al.* 2018). For the RNA modality, we filtered out the genes expressed in <200 cells. After that, 2000 highly variable genes selected by FindVariableFeatures were used for downstream analyses. For the protein modality, all proteins are used in downstream analyses. Specifically, for BMNC, BM2, SLN, and CBMC, we used 25, 134, 111, and 10 protein features, respectively. We use RunUMAP to generate UMAP (McInnes *et al.* 2018) plots in the article.

To generate clusters for the factorization of complementary information, we first use RunPCA to compute the principal components for both RNA and protein modalities. Then, we use FindNeighbors with the top 30 principal components (PCs) to construct the KNN graph for the RNA modality. For the protein modality, we use FindNeighbors with 10 PCs to construct the KNN graphs. Next, we apply FindClusters to find clusters in both modalities using the Leiden clustering algorithm (Traag *et al.* 2019).

### 2.4 Evaluation metrics

#### 2.4.1 *Metrics for clustering evaluation*

We use the normalized mutual information (NMI) and adjusted Rand index (ARI) to evaluate the clustering performance. The definition of NMI metric is as follows. Let Y and $\hat{Y}$ be the true cluster labels and the predicted cluster labels. The function I is to compute mutual entropy between two variables. The function H to compute Shannon entropy between two variables. The NMI metric is defined as,


(15) NMIY,Y^=2×IY,Y^HY+HY^#.

NMI is a real value between 0 and 1. Generally speaking, a favorable clustering result will obtain a high NMI score.

ARI is to evaluate the similarity between the clustering labels and the true labels. In theory, n data points can generate a total of $\left(\begin{array}{l} n\\ 2 \end{array}\right)$ data pairs.

The relationship of the pairwise data points can be separated into four categories. The first kind of relationship is the agreement of aggregation. That is, two data points of a pair are reported in the same cluster not only by the true labels but also by the clustering labels. The number of the aggregation agreements is denoted as a. The second kind of relationship is the agreement of separation. That is, a pair of data points that are with distinct true labels are also assigned to different clusters by the clustering labels. The number of the separation agreements is denoted as b. The rest kinds of the relationships represent the disagreement between the true labels and the clustering labels on the data pairs. Rand index (RI) defined by Equation (16) is to compute the chance that the true labels and the clustering labels will agree on a randomly chosen pair. ARI is an adjustment of RI, which is more commonly used in single-cell analysis.


(16) RI=a+bn2 #.

(17) ARI=RI-ExpectedRImaxRI-ExpectedRI#.

#### 2.4.2 *Metrics for classification evaluation*

In addition to clustering, we evaluate the multimodal single-cell integration methods by running the cell-type classification on the integrated results. We assess the classification using several metrics, including accuracy, precision, recall, F1, and Matthews’s correlation coefficient (MCC). These metrics are defined upon true positives (TP), true negatives (TN), false positives (FP), and false negatives (FN) as described in the following equations,


(18)  Accuracy=TP+TNTP+TN+FP+FN#.

(19) Precision=TPTP+FP#.

(20)  Recall=TPTP+FN#.

(21)  F1=2×Precision×RecallPrecision+Recall#.

(22) MCC=TP×TN-FP×FNTP+FPTP+FNTN+FPTN+FN#.

### 2.5 Comparison methods

We selected three methods for comparison, including Seurat V4, totalVI, and MOFA+ (Table 2). Seurat V4 provides a joint KNN graph for a given multimodal data. To visualize cell embedding, we input the KNN graph to the RunUMAP function to compute the 2D representations of cells. For clustering and classification, the 20D representations of cells are computed by using RunUMAP. Both totalVI and MOFA+ compute the embedding of cells from the given RNA and protein data straightforwardly. We used the latest release of these methods and ran with the default parameters throughout the experiments in the article.

**Table 2.** Methods for single-cell multimodal analysis.

Method	Methodology	Language	Reference
Seurat	Weighted nearest neighbor analysis	R	Hao *et al.* (2021)
totalVI	Deep generative model	Python	Gayoso *et al.* (2021)
MOFA+	Factor analysis	R	Argelaguet *et al.* (2020)

## 3 Results

### 3.1 scME leverages the shared and complementary information to improve the embedding of single-cell multiomics

**Figure 2.**
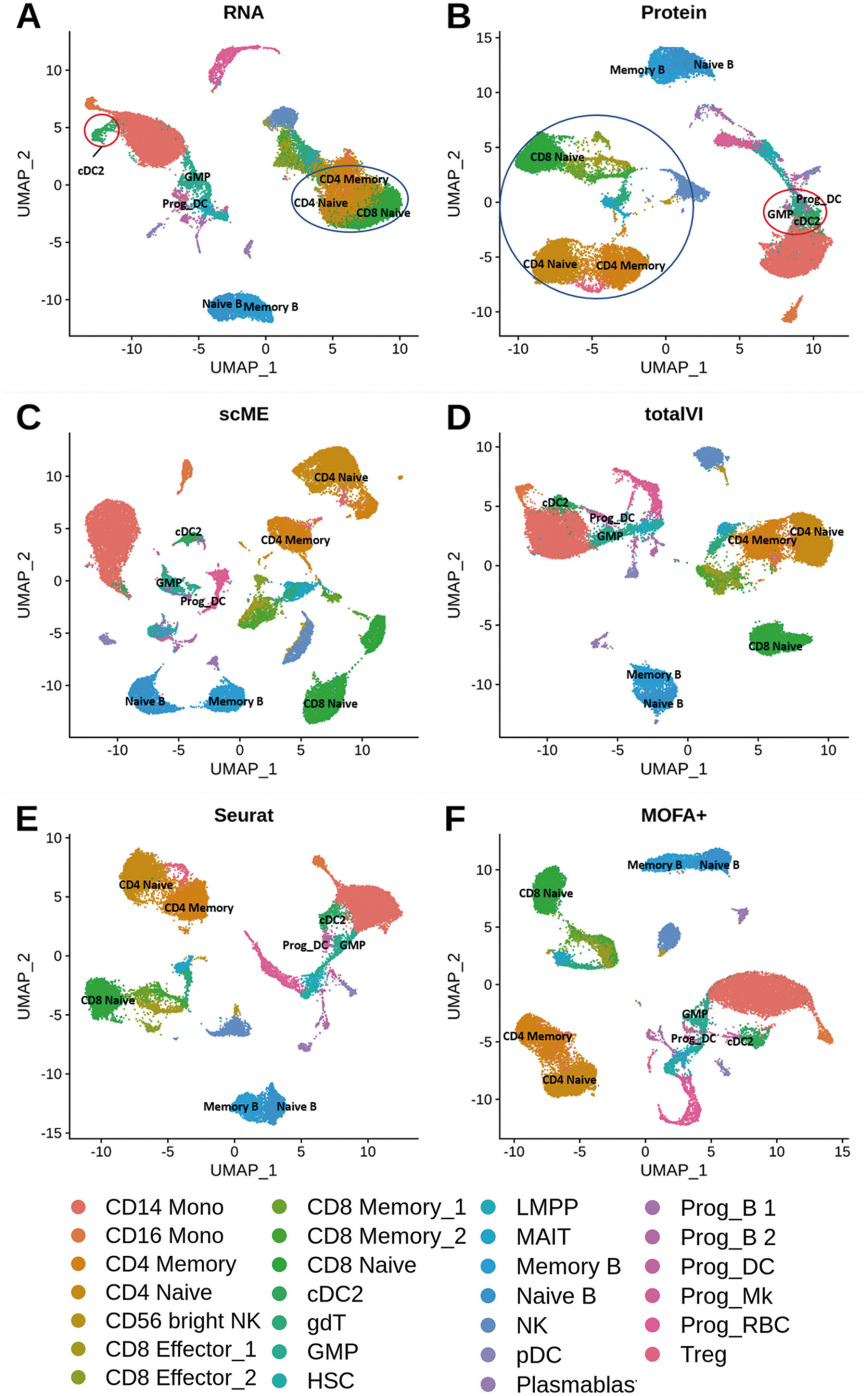
UMAP visualization of human bone marrow dataset (BMNC). (A) UMAP visualization of the RNA modality. (B) UMAP visualization of the protein modality. (C–F) UMAP visualization of the low-dimensional representations generated by scME, totalVI, Seurat, and MOFA+. All figures annotated with the labels provided by data generators.

First, we used the BMNC dataset to demonstrate the concepts introduced in the article and to show the improvements brought by scME. The embeddings generated by using the RNA modality and the protein modality separately were depicted in Fig. 2A and B. The RNA and protein modalities could provide distinct relationships among cells. It was found that most cell types were separated in both two modalities. However, there existed a few cell types that could be detected in one modality but could not be detected in the other modality. For example, the cell type cDC2 was clearly separated from the cell types GMP and Prog_DC in the UMAP visualization of the RNA modality. Contrastingly, in the protein modality, cDC2 clustered with GMP and Prog_DC. Another example of complementary information regards the clustering of the cell types CD4 Memory, CD4 Naïve, and CD8 Naïve. These T-cell subtypes were difficult to be separated by using gene expression. But the expression of the surface proteins CD4 and CD8 were such considerably different that three T-cell subtypes were separated in the protein modality.

We determined the proportion of the shared and complementary information in the BMNC dataset based on the cell–cell relationships using two different modalities. Separately for the RNA and protein modalities, we utilized the Leiden algorithm to determine the relationship between each pair of cells (Supplementary Fig. S1). If two cells are grouped into the same clusters in both modalities or are separated in both modalities, the pair is regarded as an instance of shared information. Otherwise, a pair of cells is considered to represent a single instance of complementary information. Approximately 10.5% of 470 370 456 cell pairs in BMNC were found to have inconsistent clustering relationships, indicating that the paired cells are grouped to the same clusters in one modality but to different clusters in the other modality. Consequently, it is expected that the combination of shared and complementary information will optimize the characterization of the differences among cells.

We used UMAP to display the low-dimensional multimodal embedding results generated by scME, totalVI, Seurat, and MOFA+. Overall, scME provided a multimodal embedding, where distinct cell types were clearly separated away from each other (Fig. 2C). In the multimodal embedding results of totalVI and MOFA+, it remained difficult to distinguish cDC2 from either GMP or Prog_DC (Fig. 2D and E). We also found that scME improved the characterization of nuanced cell heterogeneity in both two modalities. For example, the differences between Naïve B cells and Memory B cells were subtle in the RNA modality (Fig. 2A) as well as in the protein modality (Fig. 2B). As shown in Fig. 2C, scME could leverage the combination of different kinds of information of multiple modalities to enhance the separation of the B-cell subtypes, and eventually, to improve the identification of nuanced cell heterogeneity. In contrast, in the co-embedding results of totalVI, Seurat, and MOFA+, the B-cell subtypes with subtle molecular differences were difficult to be separated.

**Figure 3.**
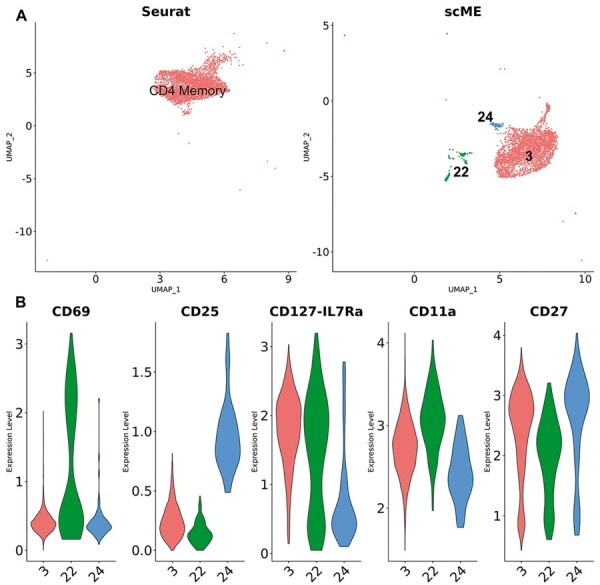
scME reveals novel cell clusters. (A) The Seurat and scME-generated UMAPs of CD4+ naïve T cells. (B) Violin plot of differentially expressed proteins.

scME revealed some tiny clusters that were not observed by other methods. Let’s consider CD4+ Memory T as an example. Compared with the result yielded by Seurat, scME divided the cells of CD4+ Memory T into one large cluster with the identity 3 and two tiny clusters recognized by the identities 22 and 24 (Fig. 3A). We conducted differential expression analysis on three clusters for the RNA and protein modalities separately (Supplementary Tables S1 and S2). The expression levels of genes were not significantly different among three clusters (Supplementary Fig. S9). But several proteins were differentially expressed, including CD69 and CD25 (Fig. 3B). This result demonstrates that scME is more effective to capture the cell-to-cell variability.

### 3.2 scME improves single-cell clustering

The above results show that scME can generate the informative joint representation of multiple modalities. But it is uncertain whether the joint representation of multiple modalities can improve single-cell clustering. To validate this, we applied scME, totalVI, Seurat, and MOFA+ to obtain the low-dimensional multimodal embedding for the four datasets. Next, we ran the Leiden clustering algorithm on the multimodal embedding results. The accuracy of single-cell clustering was assessed with the ARI and NMI metrics. We performed single-cell clustering on the RNA and protein modalities individually to provide the evaluation baseline. We also involved a vanilla auto-encoder (AE) to provide a baseline for multimodal integration. Briefly, the data of two modalities were straightforwardly concatenated and input to the vanilla AE to compute the multimodal embedding. Totally, we conducted 20 replicate experiments and reported the average values. All the experimental results were given in Tables 3 and 4.

**Table 3.** ARI statistics of single-cell clustering.^a^

Method	scME	RNA	Protein	AE	Seurat	totalVI	MOFA+
BMNC	**0.7371**	0.6596	0.6233	0.6875	0.7366	0.5433	0.5598
BM2	**0.7914**	0.5452	0.5484	0.6374	0.7741	0.5908	0.4953
CBMC	**0.7742**	0.5829	0.5142	0.6819	0.6916	0.5596	0.5446
SLN	0.7375	0.4332	0.6165	0.5341	**0.7620**	0.4588	0.5147

a The numbers in bold are the best performances.

**Table 4.** NMI statistics of single-cell clustering.^a^

Method	scME	RNA	Protein	AE	Seurat	totalVI	MOFA+
BMNC	**0.8605**	0.7301	0.7847	0.7931	0.7973	0.6789	0.7722
BM2	**0.8893**	0.7208	0.7648	0.7685	0.8610	0.7909	0.7537
CBMC	**0.8406**	0.7111	0.7936	0.7183	0.7380	0.7566	0.7153
SLN	**0.8306**	0.6039	0.7049	0.6731	0.7979	0.6719	0.6513

a The numbers in bold are the best performances.

Both ARI and NMI statistics suggested that scME obtained several advantages. First, the integration of multimodal information improved single-cell clustering. For all four datasets, scME generated more accurate clustering results in comparison with those using the unimodal feature. For example, in the results of the CBMC dataset, the ARI metrics of the clustering based on the RNA modality and the protein modality are 0.5829 and 0.5142, respectively. These results implied that the differences among cells in the CBMC dataset were subtle either in the RNA or protein modalities. scME increased the ARI metric up to 0.8742. Second, scME can make effective use of both shared and complementary information to improve clustering. Interestingly, we found that the performances of totalVI and MOFA+ were worse than that of the vanilla AE. Taking the SLN dataset as an example, both ARI and NMI statistics showed that the vanilla AE outperformed totalVI and MOFA+. Both scME and Seurat relied on the combination of various multimodal information. As expected, the results of these two methods were better than that of the vanilla AE. Encouragingly, scME was superior to Seurat in almost all four datasets except the SLN dataset. Taken together, the above results implied that scME can effectively integrate two modalities and provide informative joint representations for clustering.

### 3.3 scME improves cell-type classification

**Figure 4.**
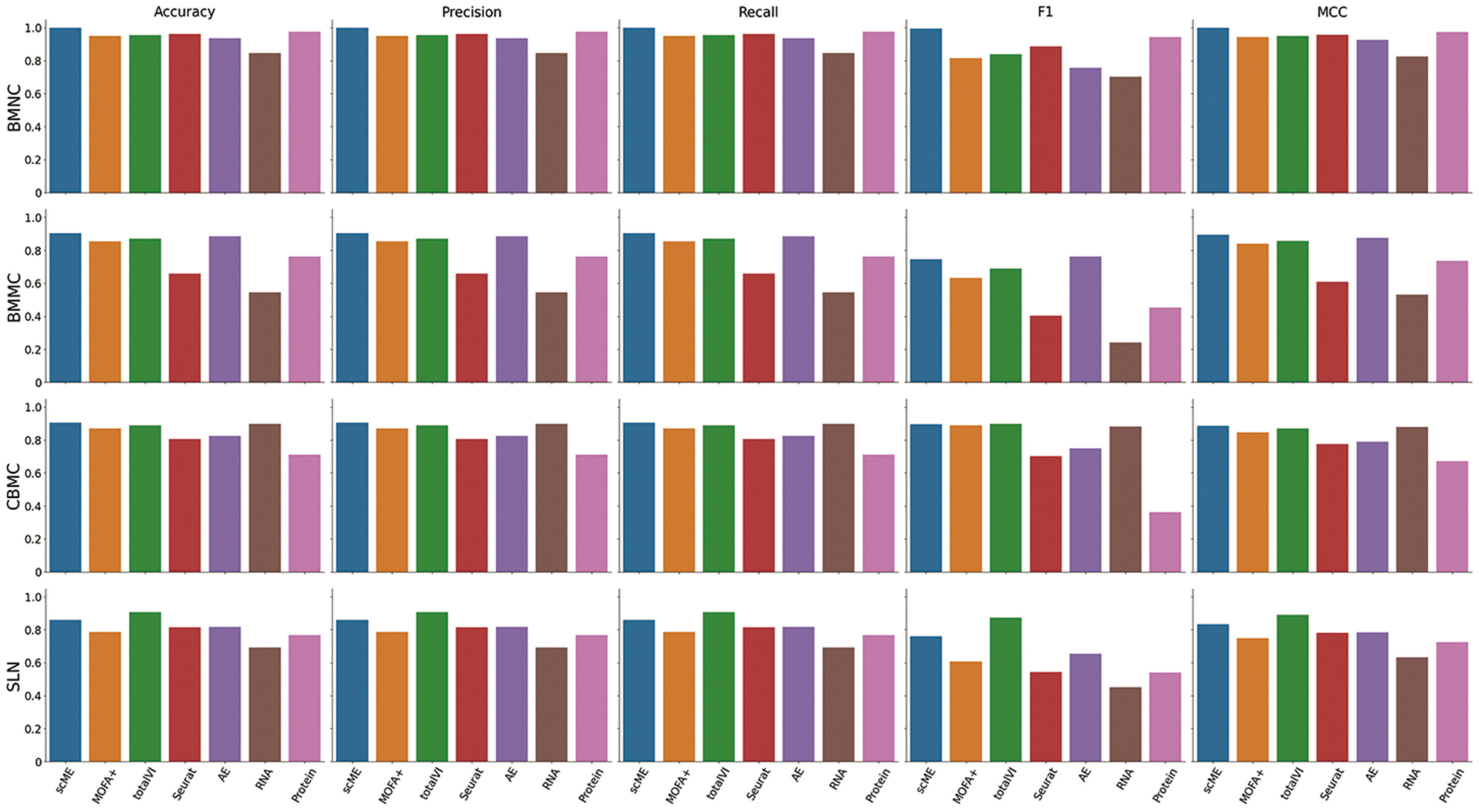
Results of the classification task. We conducted 5-fold cross-validation and computed the classification metrics including accuracy, precision, recall, F1, and MCC. Each row corresponds to the results of a dataset and each column corresponds to a metric.

Finally, we evaluated the accuracy of cell-type classification based on either the multimodal or unimodal embedding. For each embedding, a support vector machine was trained with the given cell embedding for predicting cell types. We evaluated the comparison methods by using the 5-fold cross-validation. The metrics of accuracy, precision, recall, F1, and MCC were reported in Fig. 4 and Table 5.

**Table 5.** Performance evaluation of comparison methods on cell-type classification.^a^

Dataset	Metric	scME	MOFA+	totalVI	Seurat	AE	RNA	Protein
BMNC	Accuracy	**0.9993**	0.9491	0.9556	0.9625	0.9346	0.8462	0.9766
	Precision	**0.9993**	0.9491	0.9556	0.9625	0.9346	0.8462	0.9766
	Recall	**0.9993**	0.9491	0.9556	0.9625	0.9346	0.8462	0.9766
	F1	**0.9939**	0.8142	0.8395	0.8863	0.7562	0.7027	0.9434
	MCC	**0.9992**	0.9424	0.9497	0.9575	0.9258	0.8256	0.9735
BM2	Accuracy	**0.9043**	0.8550	0.8699	0.6583	0.8853	0.5451	0.7615
	Precision	**0.9043**	0.8550	0.8699	0.6583	0.8853	0.5451	0.7615
	Recall	**0.9043**	0.8550	0.8699	0.6583	0.8853	0.5451	0.7615
	F1	0.7459	0.6337	0.6888	0.4040	**0.7630**	0.2409	0.4524
	MCC	**0.8936**	0.8413	0.8572	0.6087	0.8747	0.5305	0.7359
CBMC	Accuracy	**0.9052**	0.8700	0.8886	0.8059	0.8240	0.8982	0.7125
	Precision	**0.9052**	0.8700	0.8886	0.8059	0.8240	0.8982	0.7125
	Recall	**0.9052**	0.8700	0.8886	0.8059	0.8240	0.8982	0.7125
	F1	0.8967	0.8891	**0.8987**	0.7015	0.7490	0.8815	0.3637
	MCC	**0.8868**	0.8455	0.8692	0.7757	0.7903	0.8783	0.6708
SLN	Accuracy	0.8588	0.7868	**0.9067**	0.8139	0.8159	0.6921	0.7666
	Precision	0.8588	0.7868	**0.9067**	0.8139	0.8159	0.6921	0.7666
	Recall	0.8588	0.7868	**0.9067**	0.8139	0.8159	0.6921	0.7666
	F1	0.7603	0.6079	**0.8732**	0.5443	0.6550	0.4513	0.5407
	MCC	0.8341	0.7489	**0.8907**	0.7810	0.7835	0.6326	0.7240

a The numbers in bold are the best performances.

Overall, the results prove that scME outperformed other methods. First, scME extracted useful features from two modality data to improve the discrimination of cell types. It is noticed that the unimodal embedding, either RNA or protein, generated the worst cell-type classification results for most of the benchmarking datasets. It is interesting that the accuracy of Seurat’s multimodal embedding was worse than that of the unimodal embedding in the cases of BM2 and CBMC. As demonstrated by the results of MOFA+ and totalVI, shared information generated the robust and accurate cell-type predictions in most benchmarking datasets. Encouragingly, the combination of complementary information with shared information indeed improved the cell-type classification. scME achieved better classification results than the other methods in all metrics. We also calculated receiver operating characteristic (ROC) curves and precision–recall (P-R) curves. As can be seen from the ROC and P-R curves (Supplementary Figs S7 and S8), our method performs better than other models.

Second, scME improved the discrimination of rare cell types. It is noticed that for all benchmarking datasets, the F1 score was usually worse than the classification accuracy for all methods. Because the F1 score is defined by averaging F1 scores over cell types, this phenomenon implied that rare cell types impacted the classification accuracy. Among the comparison methods, the F1 scores of MOFA+ and totalVI declined significantly. For example, the F1 scores of MOFA+ were as low as 0.6337 and 0.6079 for the BM2 and SLN datasets, respectively. Rare cell types also impacted the accuracy of scME, but the influences were the least for most benchmarking datasets. It implied that the combination of the shared and complementary information can help the classification of rare cell types.

### 3.4 Clustering method and hyperparameter selection

**Figure 5.**
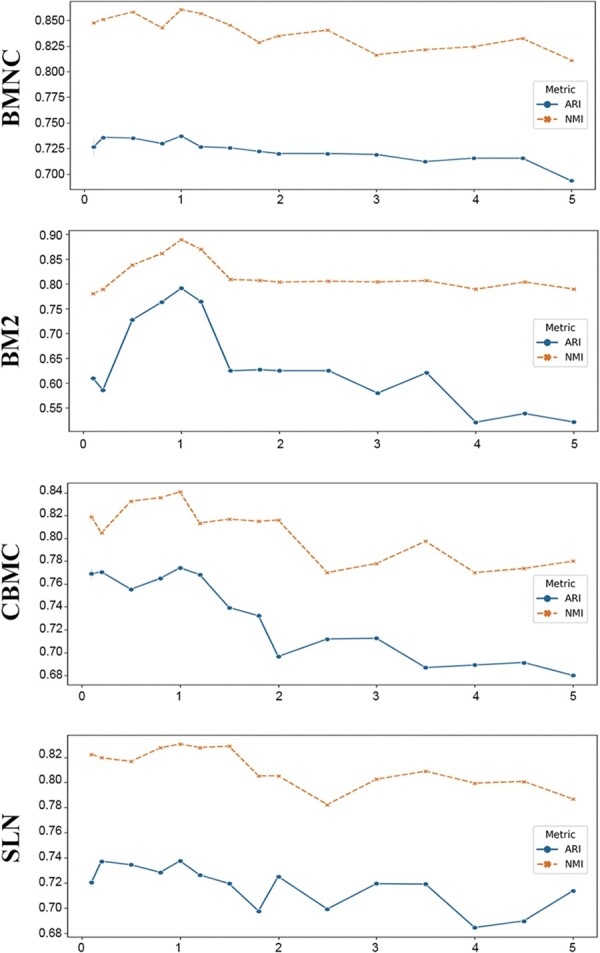
Performance of scME when using different resolution parameters of the Leiden algorithm.

Our algorithm relies on clustering to determine the shared and complementary information between modalities. Hence, the parameters of a clustering algorithm, such as resolution of the Leiden algorithm, could affect the efficacy of our algorithm. To give a reference assisting the determination of the resolution parameter of the Leiden algorithm, we assessed the impacts of a variety of resolutions by using the four datasets. The resolution of the Leiden algorithm was progressively increased from 0.1 to 5. Both ARI and NMI were calculated to evaluate the performance. Intriguingly, a common phenomenon was obtained that the resolution of 1 generated the best results for the four datasets (Fig. 5). We also compared the impacts of different clustering algorithms, including the Leiden algorithm, the KMeans algorithm, and the DBSCAN algorithm, on the performance of our method. The results demonstrate that the Leiden algorithm is more compatible with our method (Supplementary Fig. S6).

## 4 Discussion

As single-cell multiomics advance, it is urgently needed that a method can utilize the information from different modalities to maximize the dissection of cell heterogeneity. In this article, we presented a dual-modality factor model based on deep neural networks for the extraction of shared information and complementary information across various modalities. We have conducted a series of experiments and showed that combining shared and complementary information can significantly improve the joint embedding of multiple modalities. Moreover, the resultant multimodal embedding provides salient information that can dramatically improve both single-cell clustering and cell-type classification. Along with the development of single-cell multiomics, more and more single-cell modality datasets will be generated for the solution of a variety of biological and medical questions. The concept and the method of factorization proposed in this article will impact these single-cell multiomics studies. The conceptual framework of the factorization and combination of the RNA and protein modalities in CITE-seq can be extended to deal with various modalities. In the future, we will take efforts to generalize the proposed deep neural network-based factor model to involve more multimodal information for the exploration of cell heterogeneity and complex regulatory networks underlying the precise control of cell fate decisions and function.

## Supplementary Material

btad337_Supplementary_Data

## Data Availability

All datasets analyzed in this article are publicly available as listed in Table 1. The code is public for academic use and available on the GitHub site (https://github.com/bucky527/scME).
