# Supplementary material for: scME: a dual-modality factor model for single-cell multiomics embedding
Source: Bioinformatics. 2023 May 23;39(6):btad337. doi: 10.1093/bioinformatics/btad337 (PMC10234764; doi:10.1093/bioinformatics/btad337)
Supplement: btad337_Supplementary_Data [file btad337_supplementary_data.docx]

**Supplementary Materials**

#### Supplementary Figures


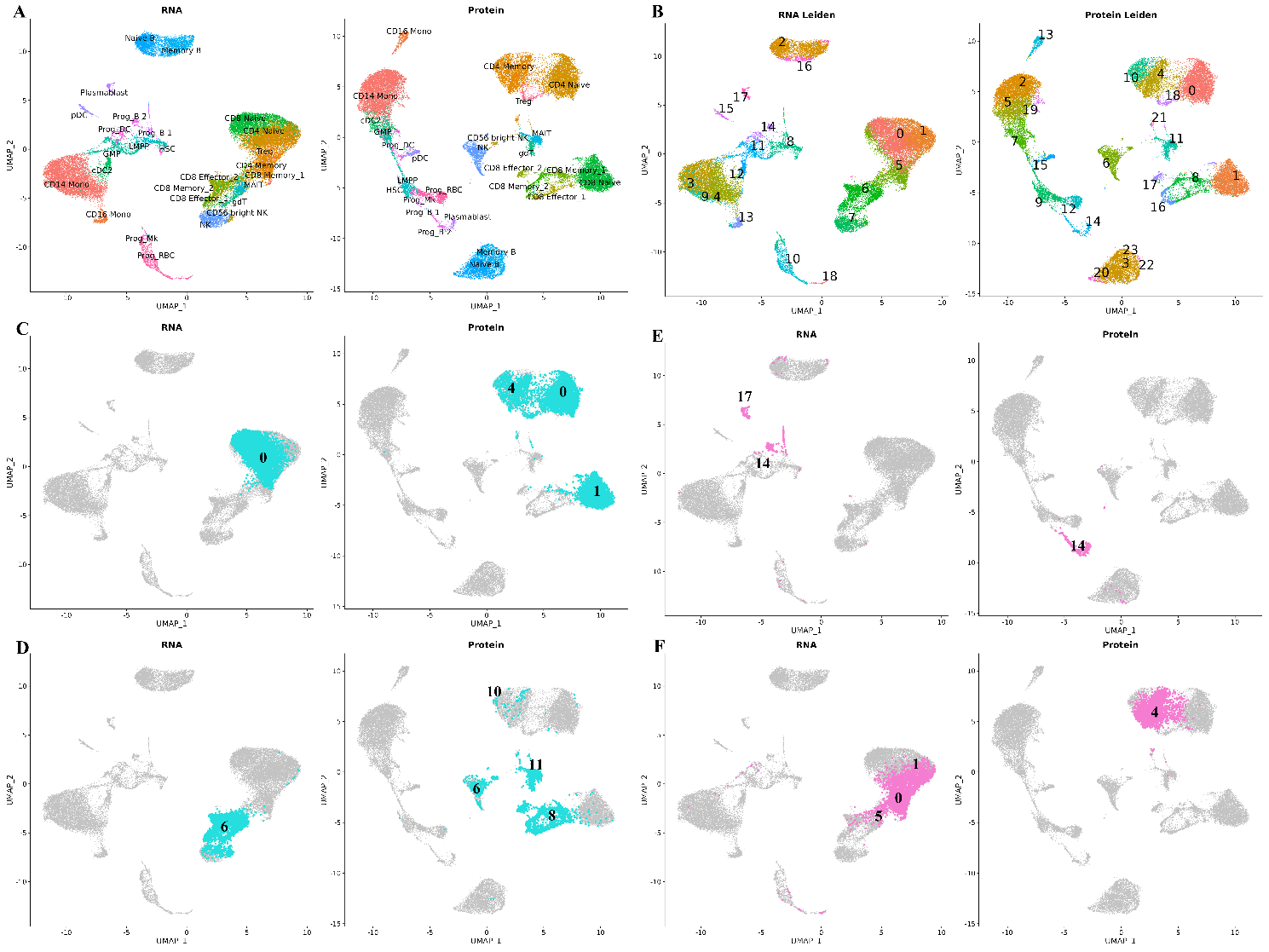
**Figure S1.** **Differences in clustering results between protein data and RNA data for BMNC data. (**A) UMAP visualization of BMNC annotated with the labels provided by data generators. (B) UMAP visualization of BMNC annotated with the Leiden clustering result. (C-D) Cells that are distinguished in proteins but grouped together in RNA. (E-F) Cells that are distinguished in RNA but grouped together in protein.


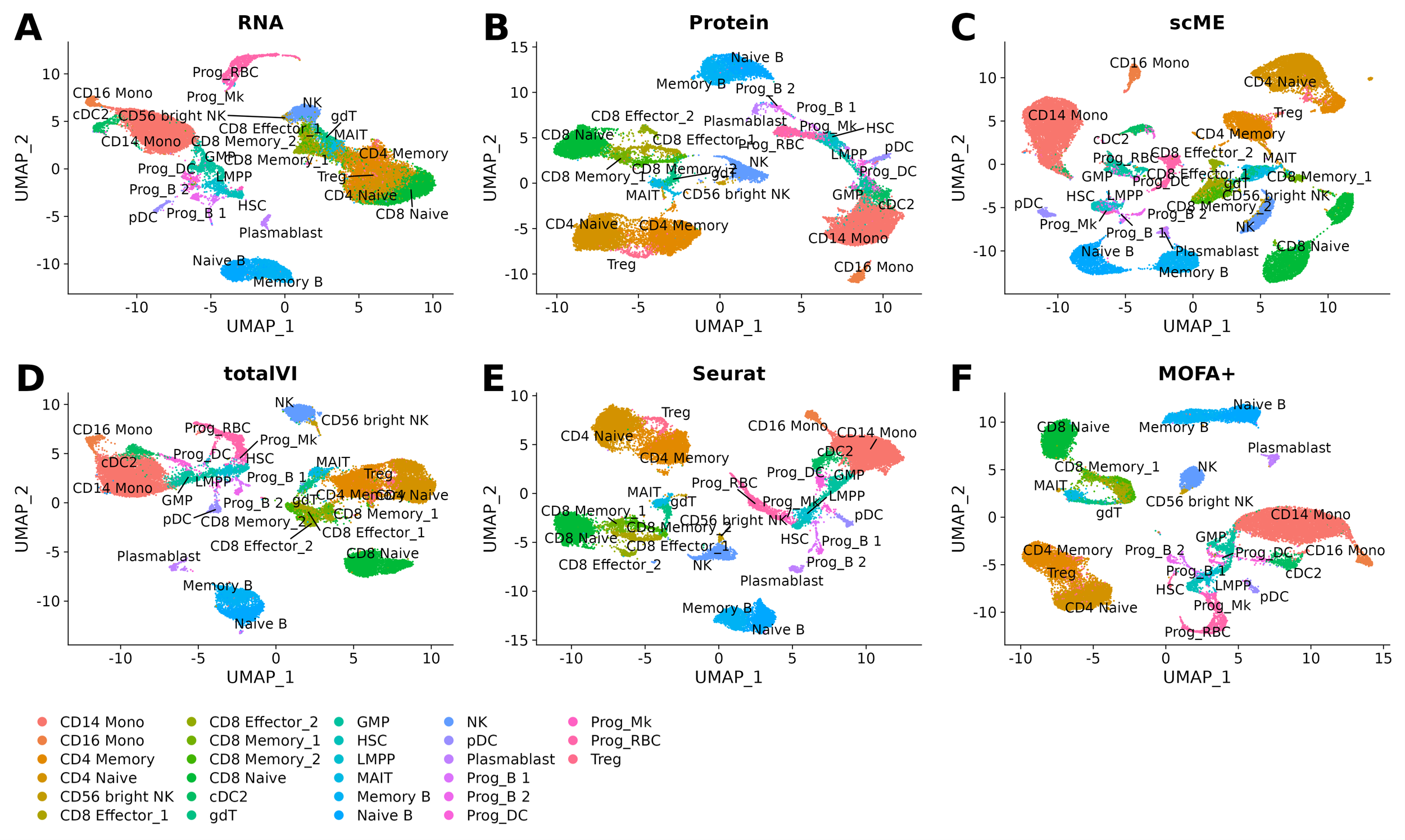
**Figure S2.** **UMAP visualization of BMNC with labels for all cell types**
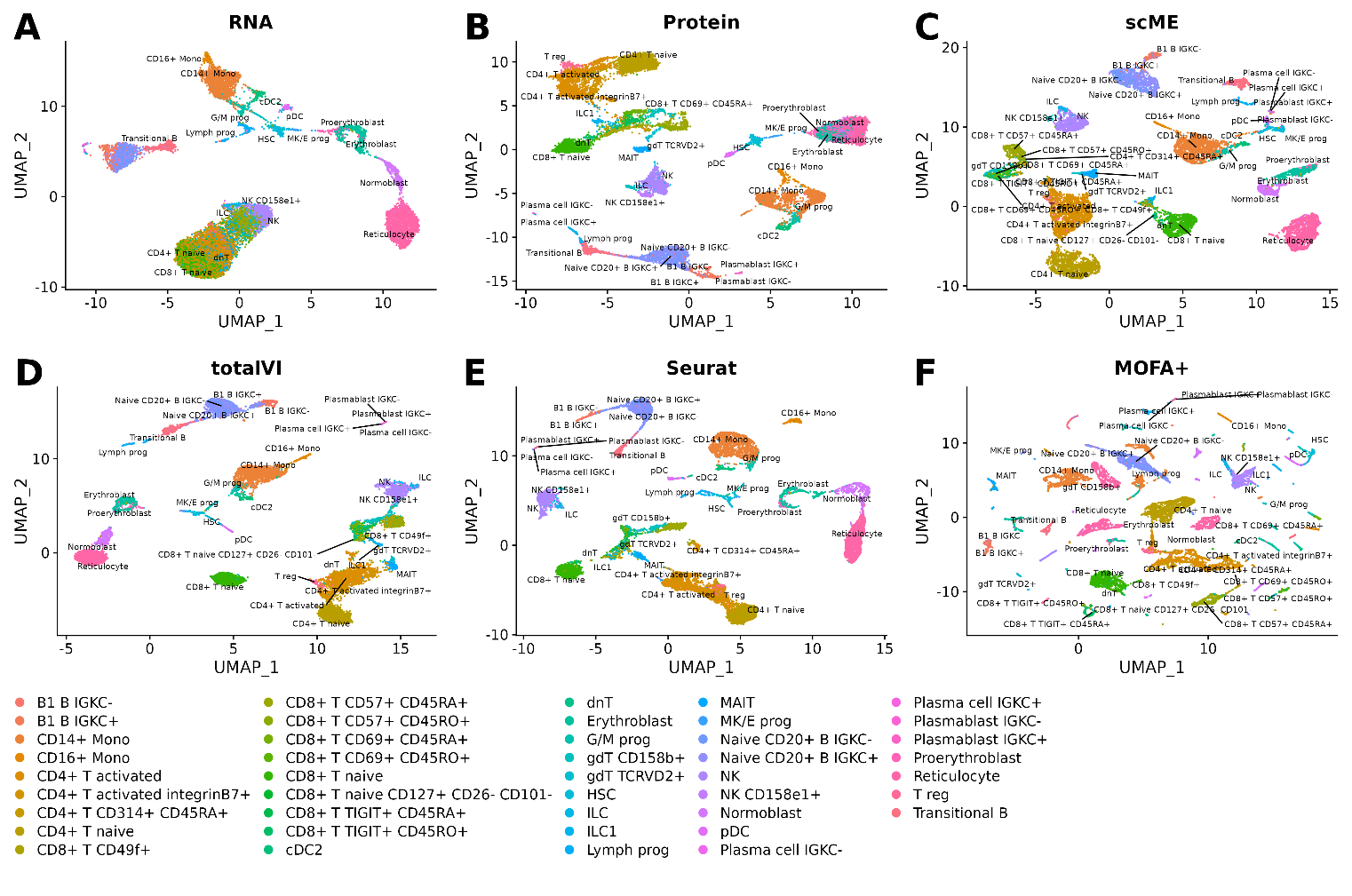


**Figure S3. UMAP visualization of BM2 with labels for all cell types**.

.


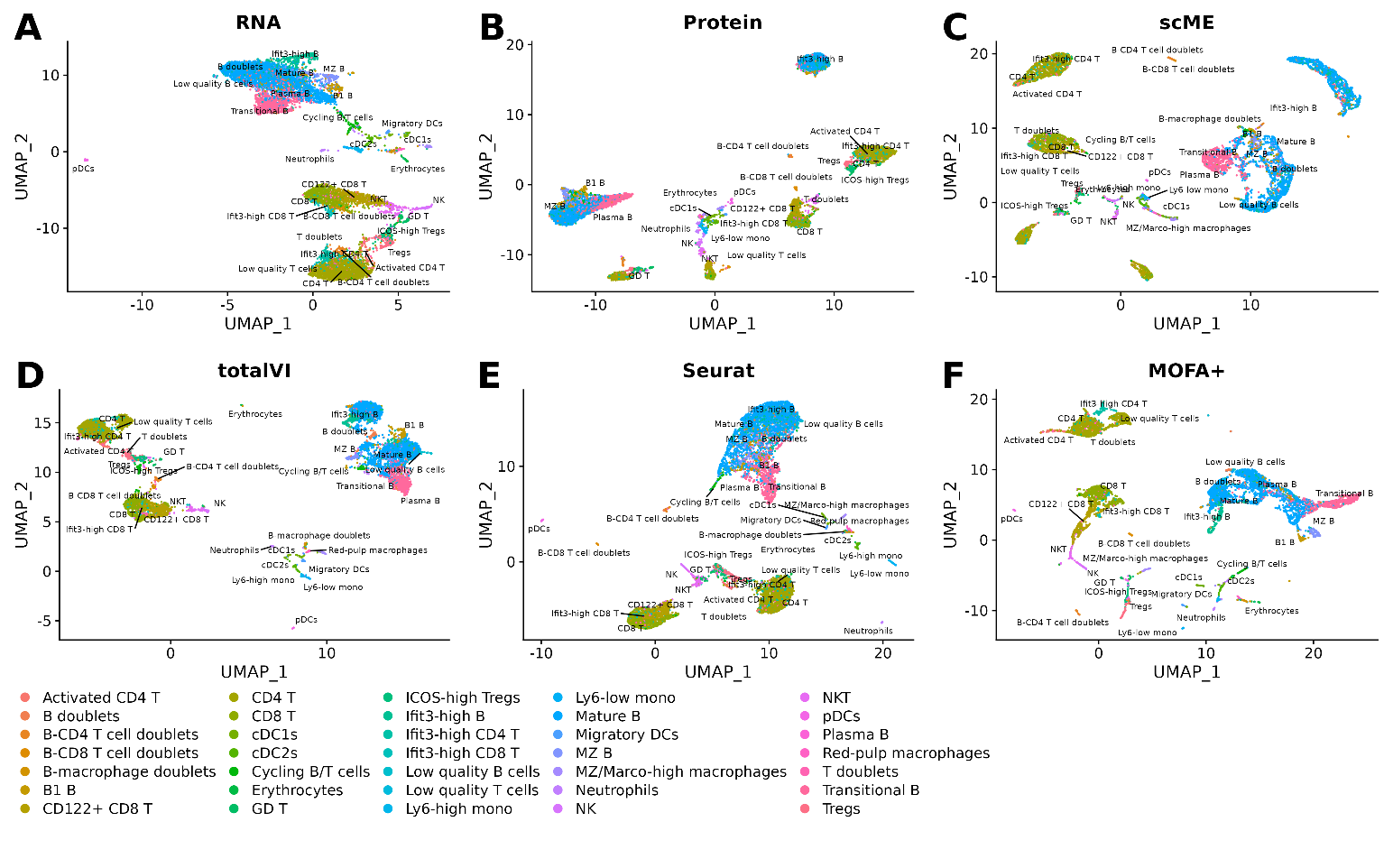


**Figure S4.** **UMAP visualization of SLN with labels for all cell types**.

.

**
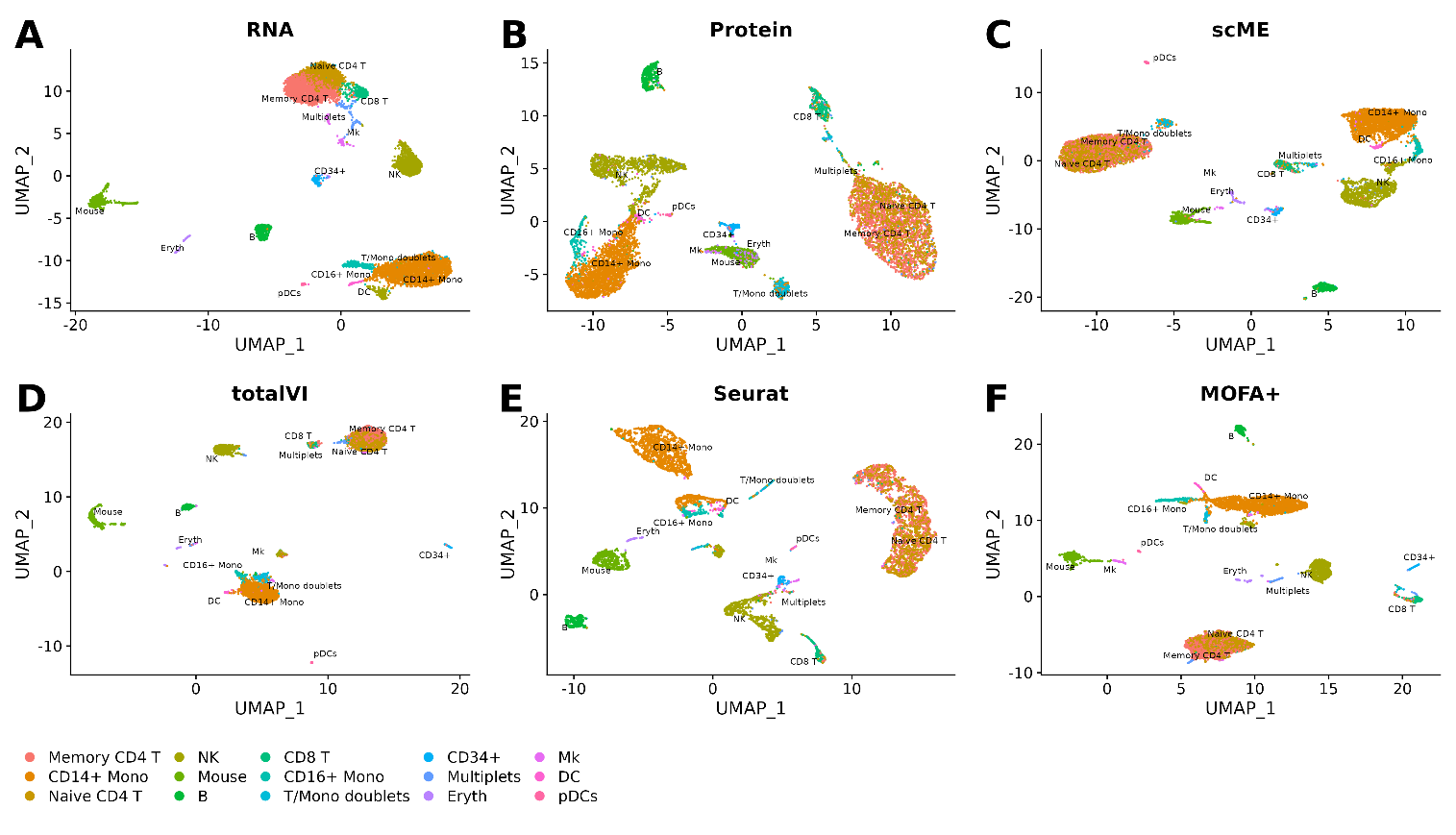
**

**Figure S5.** **UMAP visualization of CBMC with labels for all cell types**.


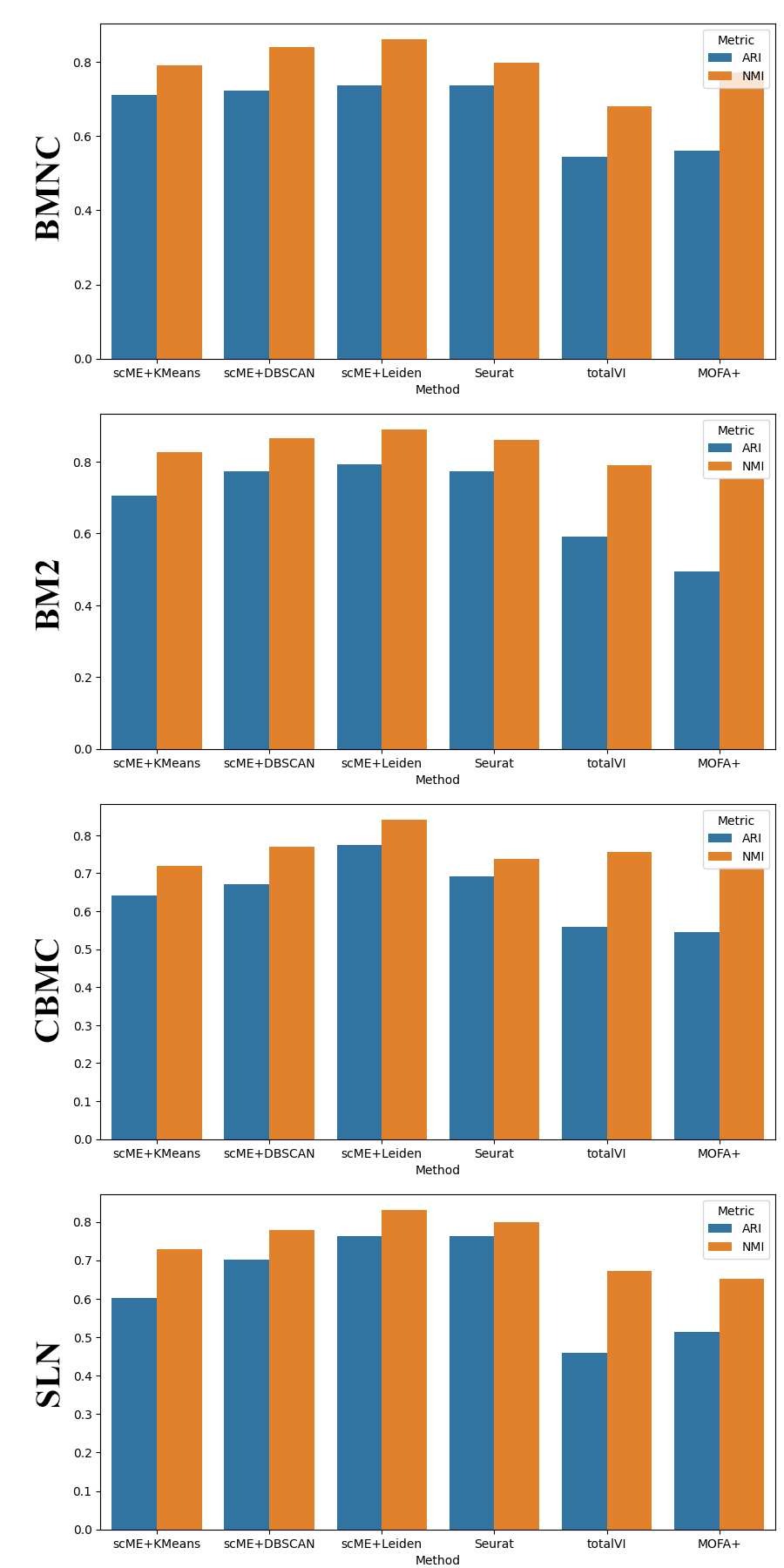


**Figure S6.** **Clustered ARI and NMI indicators using different clustering methods and other comparison algorithms in four datasets.**


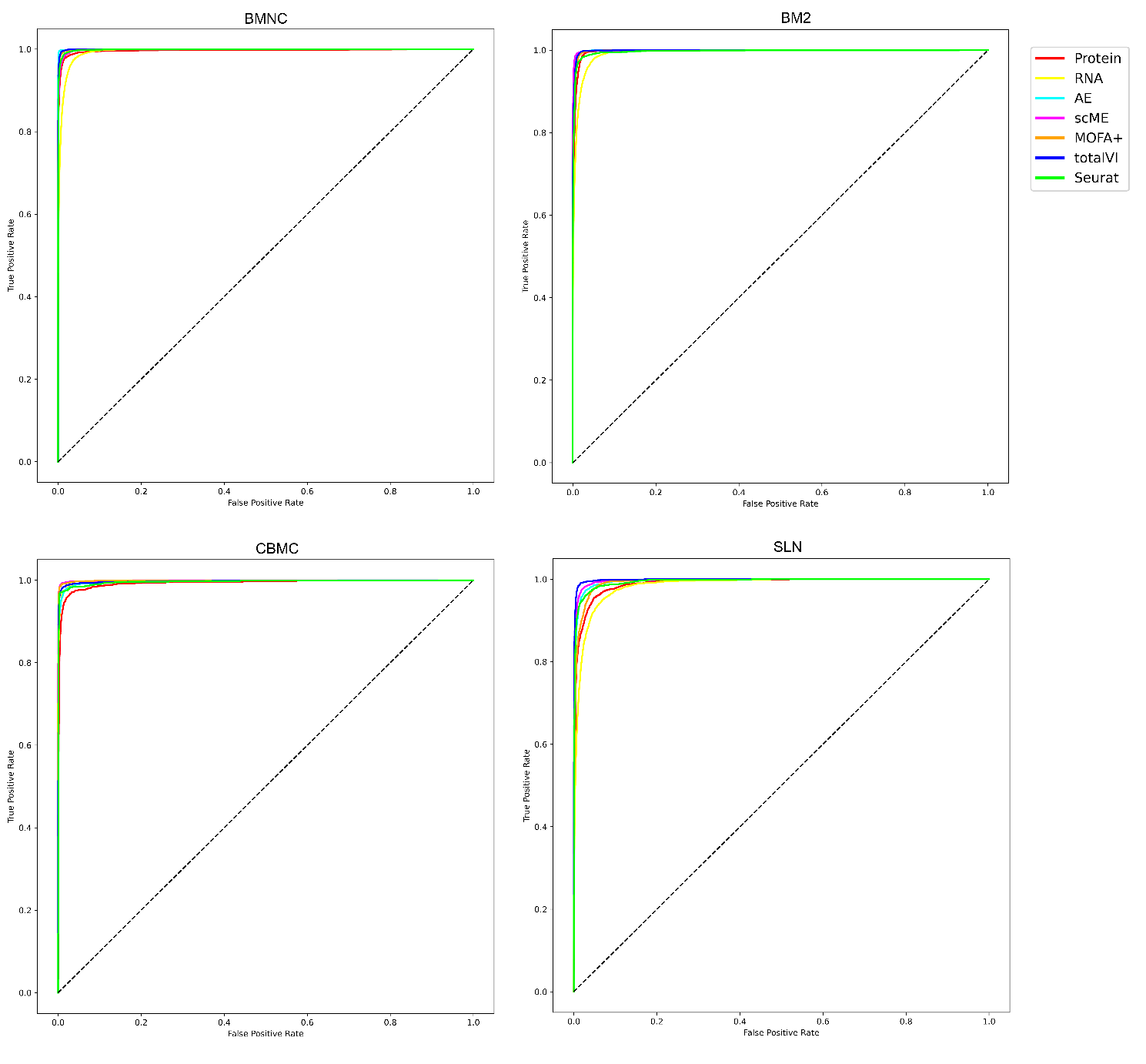


**Figure S7. ROC (Receiver Operating Characteristic) curve of comparison methods on cell-type classification.**


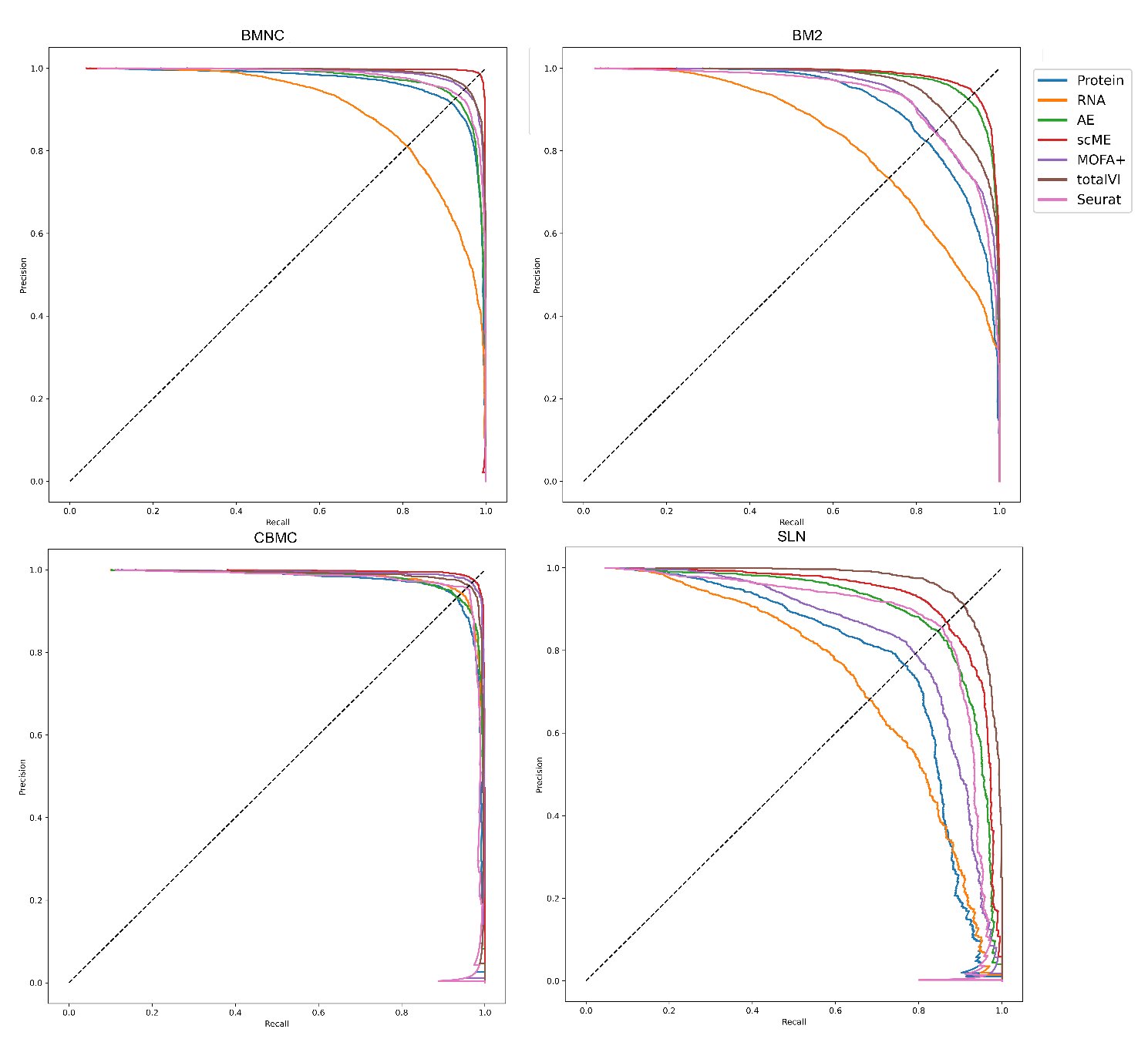


**Figure S8. P-R(Precision-Recall) curve of comparison methods on cell-type classification.**


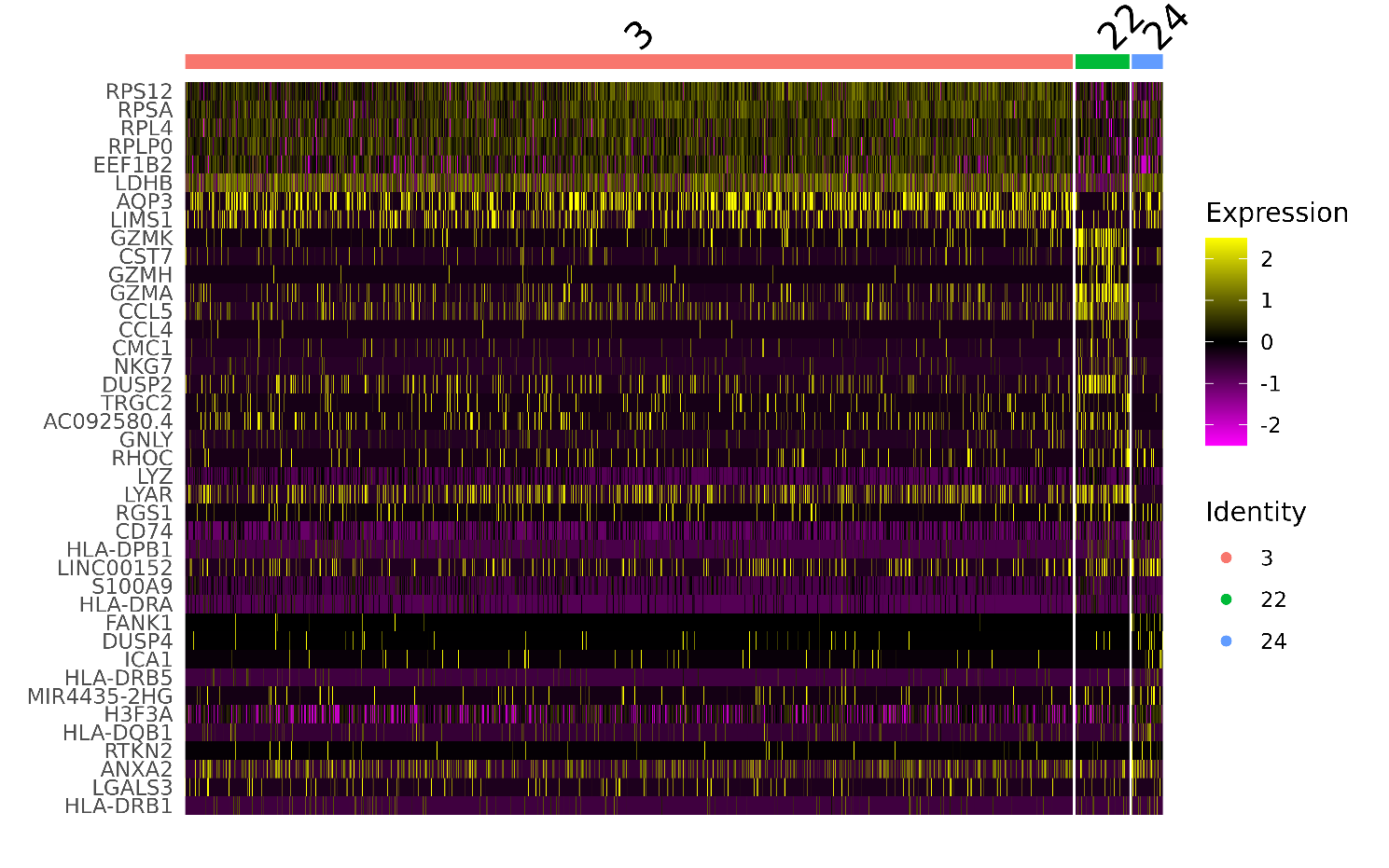


**Figure S9. Differentially expressed genes heatmap of cluster 3 , 22 and 24**

#### Supplementary Tables

**Table 1**. The differentially expressed protein between novel clusters and all other cells

| Cluster | Protein | p_val^①^ | avg_log2FC^②^ | pct.1^③^ | pct.2^④^ | p_val_adj^⑤^ |
| --- | --- | --- | --- | --- | --- | --- |
| 22 | CD45RO | 1.56E-65 | 0.5292 | 1 | 0.994 | 3.90E-64 |
|  | CD45RA | 7.92E-61 | -0.7289 | 1 | 1 | 1.98E-59 |
|  | CD4 | 3.72E-45 | 0.7125 | 1 | 1 | 9.30E-44 |
|  | CD28 | 3.93E-37 | 0.3602 | 1 | 1 | 9.83E-36 |
|  | CD278-ICOS | 3.92E-33 | 0.3125 | 1 | 1 | 9.79E-32 |
|  | CD38 | 6.57E-29 | -0.4569 | 1 | 1 | 1.64E-27 |
|  | CD11c | 1.10E-26 | -0.4593 | 1 | 1 | 2.74E-25 |
|  | CD8a | 3.30E-21 | -0.5727 | 1 | 1 | 8.26E-20 |
|  | CD127-IL7Ra | 1.03E-19 | 0.4150 | 1 | 1 | 2.57E-18 |
|  | CD123 | 3.44E-19 | -0.2571 | 1 | 0.987 | 8.61E-18 |
|  | CD3 | 4.18E-18 | 0.3908 | 1 | 1 | 1.05E-16 |
|  | CD14 | 3.25E-14 | -0.3814 | 1 | 0.999 | 8.13E-13 |
|  | CD19 | 2.25E-07 | -0.3111 | 0.994 | 0.997 | 5.63E-06 |
| 24 | CD25 | 2.61E-59 | 0.7748 | 1 | 0.966 | 6.52E-58 |
|  | CD278-ICOS | 7.45E-36 | 0.6070 | 1 | 1 | 1.86E-34 |
|  | CD28 | 8.46E-32 | 0.4624 | 1 | 1 | 2.12E-30 |
|  | CD38 | 2.30E-19 | -0.4762 | 1 | 1 | 5.74E-18 |
|  | CD4 | 3.54E-17 | 0.6246 | 1 | 1 | 8.84E-16 |
|  | CD45RA | 1.70E-16 | -0.4513 | 1 | 1 | 4.26E-15 |
|  | CD45RO | 1.85E-16 | 0.3491 | 1 | 0.994 | 4.61E-15 |
|  | CD69 | 8.65E-16 | -0.2555 | 1 | 1 | 2.16E-14 |
|  | CD11c | 2.97E-13 | -0.4505 | 1 | 1 | 7.43E-12 |
|  | CD8a | 2.51E-12 | -0.6210 | 1 | 1 | 6.29E-11 |
|  | CD14 | 1.20E-10 | -0.4166 | 0.989 | 0.999 | 2.99E-09 |
| 25 | CD278-ICOS | 2.70E-24 | 0.3848 | 1 | 1 | 6.76E-23 |
|  | CD3 | 9.31E-24 | 0.5133 | 1 | 1 | 2.33E-22 |
|  | CD27 | 3.27E-22 | 0.4708 | 1 | 1 | 8.17E-21 |
|  | CD4 | 4.44E-22 | 0.7046 | 1 | 1 | 1.11E-20 |
|  | HLA.DR | 4.11E-19 | -0.4705 | 1 | 1 | 1.03E-17 |
|  | CD28 | 2.44E-18 | 0.3458 | 1 | 1 | 6.10E-17 |
|  | CD11c | 9.91E-17 | -0.4876 | 1 | 1 | 2.48E-15 |
|  | CD127-IL7Ra | 1.76E-12 | 0.3270 | 1 | 1 | 4.41E-11 |
|  | CD123 | 7.12E-08 | -0.2500 | 1 | 0.987 | 1.78E-06 |
|  | CD14 | 2.08E-07 | -0.4092 | 0.987 | 0.999 | 5.20E-06 |

**Table 2**. The differentially expressed gene between novel clusters and all other cells

| Cluster | Gene | p_val | avg_log2FC | pct.1 | pct.2 | p_val_adj |
| --- | --- | --- | --- | --- | --- | --- |
| 22 | GZMK | 1.11E-130 | 2.2829 | 0.494 | 0.052 | 1.88E-126 |
|  | GZMA | 5.89E-54 | 1.5842 | 0.513 | 0.113 | 1.00E-49 |
|  | CCL5 | 6.67E-49 | 1.1807 | 0.617 | 0.16 | 1.13E-44 |
|  | IL32 | 3.40E-48 | 1.5725 | 0.87 | 0.396 | 5.77E-44 |
|  | B2M | 1.68E-45 | 0.7887 | 1 | 0.999 | 2.86E-41 |
|  | TRAC | 2.13E-40 | 1.3202 | 0.864 | 0.402 | 3.63E-36 |
|  | CD2 | 5.31E-33 | 1.4985 | 0.552 | 0.212 | 9.03E-29 |
|  | CST7 | 2.34E-27 | 0.7142 | 0.442 | 0.129 | 3.99E-23 |
|  | DUSP2 | 3.24E-23 | 1.1141 | 0.383 | 0.125 | 5.51E-19 |
|  | MALAT1 | 4.36E-23 | 0.5973 | 1 | 1 | 7.42E-19 |
|  | KLRB1 | 9.12E-23 | 1.4265 | 0.448 | 0.164 | 1.55E-18 |
|  | AC092580.4 | 1.85E-21 | 0.9177 | 0.266 | 0.07 | 3.14E-17 |
| 24 | FOXP3 | 0 | 0.7744 | 0.169 | 0.001 | 0 |
|  | DUSP4 | 1.81E-177 | 0.9225 | 0.18 | 0.003 | 3.07E-173 |
|  | FANK1 | 2.01E-121 | 0.6111 | 0.112 | 0.002 | 3.42E-117 |
|  | IL2RA | 7.19E-90 | 0.9467 | 0.213 | 0.009 | 1.22E-85 |
|  | HPGD | 1.83E-65 | 1.1871 | 0.236 | 0.015 | 3.11E-61 |
|  | IL32 | 1.91E-36 | 1.9385 | 0.91 | 0.397 | 3.25E-32 |
|  | RP11-1399P15.1 | 1.47E-32 | 0.8334 | 0.124 | 0.008 | 2.50E-28 |
|  | ARID5B | 1.35E-25 | 1.0842 | 0.371 | 0.076 | 2.29E-21 |
|  | TIGIT | 6.96E-22 | 0.7708 | 0.18 | 0.024 | 1.18E-17 |
|  | ITGB1 | 4.15E-21 | 1.4452 | 0.551 | 0.195 | 7.06E-17 |
|  | CD52 | 7.41E-21 | 1.0693 | 0.955 | 0.773 | 1.26E-16 |
|  | B2M | 1.32E-20 | 0.6686 | 1 | 0.999 | 2.25E-16 |
|  | LAIR2 | 1.21E-19 | 0.6950 | 0.169 | 0.023 | 2.06E-15 |
|  | STAM | 1.58E-18 | 0.5660 | 0.157 | 0.022 | 2.68E-14 |
|  | GATA3 | 1.73E-18 | 0.9552 | 0.247 | 0.048 | 2.94E-14 |
|  | ICA1 | 2.37E-18 | 0.7174 | 0.146 | 0.019 | 4.03E-14 |
| 25 | ACTN1 | 3.27E-21 | 1.1558 | 0.427 | 0.1 | 5.56E-17 |
|  | LAT | 2.09E-19 | 1.0364 | 0.627 | 0.2 | 3.56E-15 |
|  | RPS3A | 2.53E-16 | 0.4661 | 1 | 0.997 | 4.31E-12 |
|  | RPS12 | 5.06E-16 | 0.4962 | 1 | 0.998 | 8.60E-12 |
|  | RPL31 | 7.82E-16 | 0.5608 | 1 | 0.989 | 1.33E-11 |
|  | NOSIP | 1.93E-15 | 0.9266 | 0.8 | 0.39 | 3.29E-11 |
|  | LEF1 | 2.50E-15 | 0.9119 | 0.48 | 0.149 | 4.25E-11 |
|  | RPS15A | 3.20E-15 | 0.4456 | 1 | 0.997 | 5.45E-11 |
|  | RPS25 | 7.14E-15 | 0.5199 | 1 | 0.992 | 1.22E-10 |
|  | RPL32 | 3.40E-14 | 0.4007 | 1 | 0.998 | 5.79E-10 |

1. p_val: p value (unadjusted)
2. avg_log2FC: log fold-change of the average expression between the two groups. Positive values indicate that the feature is more highly expressed in the first group.
3. pct.1: The percentage of cells where the feature is detected in the novel clusters
4. pct.2: The percentage of cells where the feature is detected in the other cells
5. p_val_adj: Adjusted p value, based on Bonferroni correction using all features in the dataset.
